# Human and space ethology as a methodology-based solution for interdisciplinary research

**DOI:** 10.3389/fphys.2025.1599005

**Published:** 2025-06-26

**Authors:** Carole Tafforin, Michel Tognini

**Affiliations:** ^1^ Ethospace, Research and Study Group in Human and Space Ethology, Toulouse, France; ^2^ Astronaut from the European Space Agency, Paris, France

**Keywords:** behavior, physiology, cognition, mechanisms, adaptation, countermeasures, innovations, space exploration

## 1 Introduction

Ethology refers to the term behavioral biology as part of the life sciences. When it comes to humans in space, this discipline represents the full scope and evolution of a specific methodology whose feasibility has been demonstrated in field research. The very first example was the effect of microgravity on the astronaut’s motor behavior during short-term missions. We have compiled a repertoire of movements, postures, orientations, and positions developed objectively for the observation, description, and quantification of behavioral manifestations. They are the three specific tools used to investigate the relationship between the individual and his/her environment. Current examples include the effect of confinement and isolation on social behavior during extended periods of time simulating missions to the Moon and Mars (Tafforin, 2022). The behavioral repertoire has been expanded to actions, interactions, expressions, and communications. It allows research and study of global human adaptation to a wide range of environments and analogs such as parabolic flights, space shuttles, orbital stations, Arctic expeditions, Antarctic bases, and confinement campaigns (Tafforin et al., 2023). The results showed that human and space ethology is at the interface of a panel of disciplines, with a common topic of how the organism’s components work together to maintain a healthy state.

**FIGURE 1:**
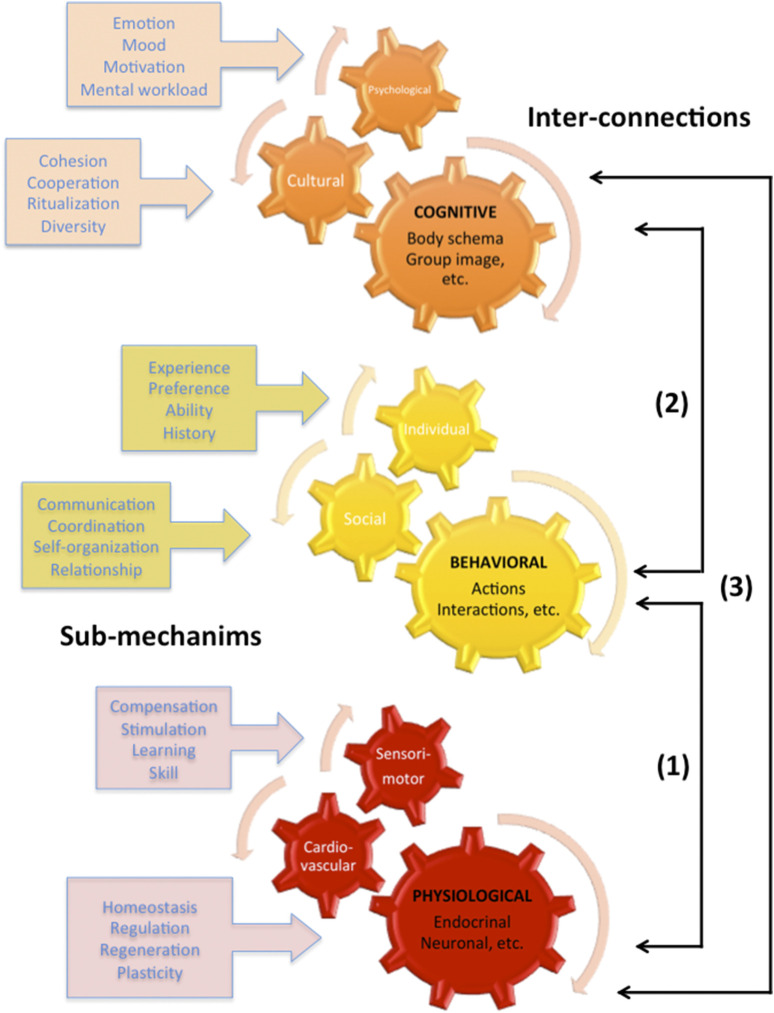
Key human mechanisms for interdisciplinary research.

The Nobel Prize in Medicine or Physiology was awarded to Ivan Petrovitch Pavlov for his work on conditioned reflexes (Pavlov, 1927). There were seminal examples of how to study the connections between a behavioral action (e.g., eating), a sensory signal (e.g., bell ringing), and a biological response (e.g., salivation). This emphasized interdisciplinary nodes, such as connections between physiology (metabolic parameters) and behavior (motor actions). Major human mechanisms, be they physiological, behavioral, or cognitive, should be studied using methods and tools specific to each discipline. The goals are to adopt a systemic approach and conduct a holistic analysis of exhaustive research data. Figure 1 illustrates the different components as wheels, with sub-mechanisms and interconnections.

(1) The physiological–behavioral connection is related to the endocrinal, neural, metabolic, vestibular, muscular, and primitive functions that underlie the behavioral emergence in terms of motor skill and motor performance. The ethological observations can be made at the physiological time scale since the level of precision is less than one second in the encoding of state and point events from video recordings.
(2) The behavioral–cognitive connection is related to body schema and group image, resulting from cognitive mechanisms that interact with behavioral activities, such as actions at the individual scale and interactions at the social scale. The ethological observations through quantitative descriptions of the items from the astronaut’s repertoire can account for cultural cohesion, emotional workload in daily life activities, and scientific and operational activities in international space missions.
(3) The physiological–cognitive connection is related, with advanced technologies and new medical developments, to correlations of data collected from both cerebral activities, cognitive tasks, and the whole battery of psychological tests. The ethological observations can help in the quantitative description of behavioral indicators (e.g., collateral actions, facial expressions, body orientations, and postural changes) that predict etiological factors as assumed in space medicine. The goal is to corroborate physical, mental, and behavioral health.

Are the measuring tools effective for all these interfaces? Each discipline has assessment methods and measurement devices, but they do not take into account the human being in its interconnected mechanisms for measuring integrative functions. In space, at the cardio-vascular level, the lack of gravitational force induces a blood shift that generates increased cardiac output and venous flow, stimulates endocrine receptors, and returns metabolic regulation to its original state. At the same time, at the sensorimotor level, microgravity induces modifications in the inputs from visual, proprioceptive, musculo-articular, and cutaneous receptors. Cognitive responses and behavioral strategies are also being built. It requires a methodology-based solution for such interplanetary research.

## 2 Systemic approach

The components and mechanisms involved in human adaptation within the framework of space life sciences are the outcomes of a systemic approach. An ethological monitoring of the astronaut’s behavioral activities allows for highlighting links between systems in an integrated model. Such a systemic approach showed connections between a hard system, which functions just like conservative regulations in the physiological sphere to restore homeostasis, and a soft-system, which functions just like innovative regulations in the motor behavior sphere to create new strategies (Custaud et al., 2020). At the upper cognitive sphere, an informative system would be used just like initial or final integrations that provide dynamic outputs about the links between the other two systems. A longitudinal and observational method is an appropriate solution in the search for multi-modal changes over time during long-term exploration missions.

A comprehensive example is confinement under Mars-like conditions, including a closed-loop system, namely, a controlled ecological life-support system (CELSS) (Yuan et al., 2019). The multi-system adaptation study was unique in its design over a 180-day experiment. On one hand, body composition, cardiovascular state, metabolic state, endothelial state, and muscle state were investigated using biological functional and morphological measurements. On the other hand, recovery-stress state and positive or negative mood were assessed through psychological questionnaires. At the interfaces, behavioral activity through ethological observations revealed interrelationships between the systems evidenced for a particular subject. Results showed the greatest collateral acts-to-facial expressions, suggesting a shift in the balance between stressful conditions and wellbeing state toward stress, along with the lowest cardio-vascular fitness and the highest losses in lean mass and body water.

An integrative example is also found during a 370-day confinement experiment of crews in the high-closure mission called “Lunar Palace 365” (Fu et al., 2025). It supported advanced innovations in biomedical engineering, environmental biology, and life support technology (e.g., optimizing closure—reaching 98.2% of materials being recycled and regenerated for human long-term survival in a lunar-like environment). From individual survivability, based on biological needs, to social adaptability, based on behavioral requirements, a systemic approach is relevant.

## 3 Holistic analysis

Currently, challenging missions aboard the International Space Station (ISS) reach a year-long exposure to microgravity, cosmic radiation, confinement, isolation, and other less-studied epigenetic factors. The Space Twins study (Garrett-Bakelman et al., 2019) offered this opportunity. The multi-omic, molecular, physiological, and behavioral datasets provided a valuable roadmap for future studies. Changes described in this multidimensional analysis serve as a guide for targeted countermeasures and monitoring during future crewed missions.

From the perspective of deep space exploration, the idea would be to progress from multidisciplinary contributions (Blackwell Landon et al., 2019) to interdisciplinary research, incorporating holistic analysis (i.e., treating the human being as a whole in their physical, mental, social, cultural, and environmental aspects within an ecosystem) with exhaustive and multimodal data. A final goal would be outcomes such as behavioral health and wellbeing of space explorers for the success of their missions. Fundamental issues of adaptation and countermeasures have been reported, and research gaps have been identified (Pagnini et al., 2023). Questions are, for instance, as follows: what variables capture relevant information on behavior and performance? What physiological biomarkers provide valid insight into the psychological experience of the astronauts? What methods, measures, and metrics should be used to monitor crewmembers and crew functions?

Solutions could be international standard measures such as those conducted during the Artificial Gravity Bed Rest with European Space Agency (AGBRESA) study (Clément et al., 2022). Simultaneous video recordings for ethological encoding, as recurrent protocols, could enrich space databases for the scientific community. In addition, collaborative system usability of audio recordings, by tracking conversations, may serve to assess workload in remote environments (Shelat et al., 2024). Similarly, the combination of cardiovascular measures and speech signals, as demonstrated in the aeronautic environment (Magnusdottir et al., 2022), would complement a holistic analysis.

## 4 Innovative applications

**FIGURE 2:**
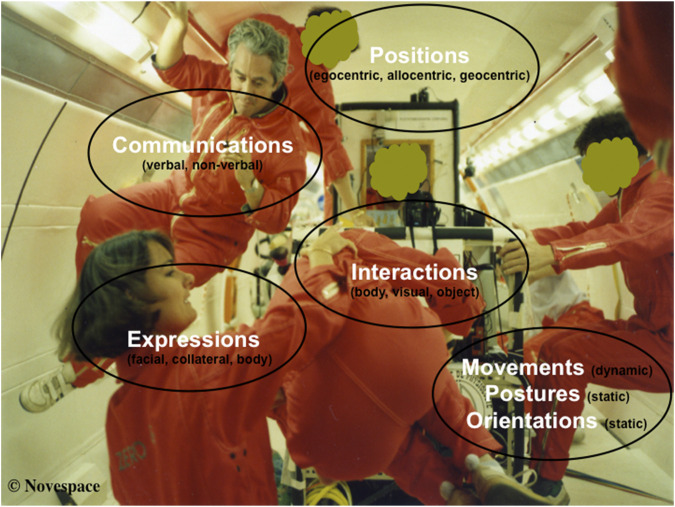
Behavioral categories observed in human and space ethology (parabolic flight).

In the space domain, there are devices applied at the personal scale, such as the Facial Action Coding System (FACS) for collecting data on crewmembers’ emotions (Ekman and Friesen, 1978), and at the interpersonal scale, such as the Wireless Group Structure (WLGS) module for collecting data on crew cohesion (Johannes et al., 2015) or sociomapping (Sykorova et al., 2024). Innovative applications could be a non-invasive “all-in-one” and “all-at-once” tool to collect behavioral data in the categories of expressions (vs. face reader), interactions (vs. sociometer), movements–postures–orientations (vs. actimeter), positions (vs. geolocalized tracking), and communications (vs. audiometer) (Figure 2). Such human and space ethology as a methodology-based solution would contribute to the concept of an *ethometer*. In the control room of a manned space mission, one could imagine joint areas of teleoperation (e.g., ultrasound images) (Greaves, 2019) and ethometric information (e.g., rate of behavioral occurrences) in live or offline video observations.

This could lead to innovations in the validation of new countermeasures and support for astronauts on deep space missions. Virtual reality technologies, voice assistants, and digital companions are the prospects (Gushin et al., 2021). Validation and corroboration of real and virtual behaviors are the resulting prospects (Tafforin, 2024). Comparisons could be made with digital twins. Human factors are also concerned with the detection of preventable human errors for mission success (Pasquier et al., 2019). A particular focus is on wellbeing and life-support on the Moon and beyond (Tafforin and Tamponnet, 2025). Methodological applications with respect to the interdisciplinary research of countermeasures, such as greenhouses in future space habitats, will help in creating an Earth-like micro-environment that supports a micro-society functioning in social self-organization and vital self-resources.

In order to promote further advances in space, along with applications on Earth, research needs continued sharpening in several areas. An example would be multi-analyses of astronauts’ body orientation to find out the disorientation processes involved in Alzheimer’s disease. Corroborated magnetic resonance images and cognitive tasks in the cerebral plasticity (Romand-Monnier, 2019) using the ethological approach would be a useful methodology-based solution to better understand behavioral adaptation.

## 5 Discussion

Scientific openings are optimistic opinions aimed at expanding the frontiers between academic activities and logistic developments, thus leading to novel cooperative programs. A realistic opinion is to remain attentive to maintain the bases of life sciences at the heart of the concrete space research program. Human mechanisms are complex, and the human observer has an irreplaceable potential as a renewing solution to understand them.

Astronautic recommendations emphasize the importance of soft skills for manned missions because living and working in confined spaces under pressure and in weightlessness requires much more than technical skills. Space agencies are looking for candidates with exceptional interpersonal and psychological abilities. We do not forget task skills, but we do include soft skills according to several aspects defined in the Human Behavior and Performance (HBP) competency model (NASA report, 2008).

Key behavioral skills sought in astronauts include the following: (a) stress and pressure management: the ability to handle high-tension situations, such as a system failure or a medical emergency, and control of emotions to avoid impulsive reactions. (b) Teamwork and collaboration: adaptability to multicultural group dynamics; clear and effective communication; and the ability to manage conflicts constructively. (c) Autonomy and decision-making: the ability to solve problems quickly and effectively in isolated situations and taking initiative while respecting hierarchy and procedures. (d) Resilience: tolerance to solitude and the monotony of long missions; resistance to extreme conditions (isolation, confinement, and lack of contact with family); and ability to stay motivated and focused over the long term. (e) Adaptability and rapid learning: flexibility in coping with unforeseen events and mission changes and willingness to learn and constantly train for new situations.

Selection and training of astronauts are major concerns, including the following: (a) extensive psychological tests to assess resilience, stress management, and team compatibility. (b) Crisis simulations to observe reactions under pressure. (c) Experiments in extreme conditions (polar bases, underground and underwater stays, and closed chambers) to evaluate the ability to live in inhospitable environments.

Challenges for exploration missions will require combined tools and methods to assess all these behavioral markers and biomarkers. Social sciences and life sciences are coming together for the future of humans in space.
